# Morphological and Photosynthetic Pigment Screening of Four Microgreens Species Exposed to Heavy Ions

**DOI:** 10.3390/plants13243541

**Published:** 2024-12-19

**Authors:** Chiara Amitrano, Sara De Francesco, Marco Durante, Walter Tinganelli, Carmen Arena, Veronica De Micco

**Affiliations:** 1 Department of Agricultural Sciences, University of Naples Federico II, 80055 Naples, Italy; chiara.amitrano@unina.it (C.A.); sara.defrancesco@unina.it (S.D.F.); 2 Biophysics Department, GSI Helmholtzzentrum für Schwerionenforschung, 64291 Darmsatdt, Germany; marco.durante@unina.it (M.D.); w.tinganelli@gsi.de (W.T.); 3 Institute for Condensed Matter Physics, Technische University Darmstadt, 64289 Darmstadt, Germany; 4 Department of Physics “Ettore Pancini”, University Federico II, 80126 Naples, Italy; 5 Department of Biology, University of Naples Federico II, 80126 Naples, Italy

**Keywords:** carbon ion, ionizing radiation, iron ion, microgreens, photosynthetic pigment content, space biology

## Abstract

Numerous challenges are posed by the extra-terrestrial environment for space farming and various technological growth systems are being developed to allow for microgreens’ cultivation in space. Microgreens, with their unique nutrient profiles, may well integrate the diet of crew members, being a natural substitute for chemical food supplements. However, the space radiation environment may alter plant properties, and there is still a knowledge gap concerning the effects of various types of radiation on plants and specifically on the application of efficient and rapid methods for selecting new species for space farming, based on their radio-resistance. Thus, the hypotheses behind this study were to explore the following: (i) the pattern (if any) of radio-sensitivity/resistance; and (ii) if the morphological parameters in relation with pigment content may be a feasible way to perform a screening of radiation responses among species. To perform this, we irradiated dry seeds of basil, rocket, radish, and cress with iron (^56^Fe; 1550 MeV/(g/cm²)) and carbon (^12^C; 290 MeV/u, 13 keV/µm) heavy ions at the doses of 0.3, 1, 10, 20, and 25 Gy to investigate the growth responses of microgreens to acute radiation exposure in terms of morphological traits and photosynthetic pigment content. Results indicate that the microgreens’ reaction to ionizing radiation is highly species-specific and that radiation is often sensed by microgreens as a mild stress, stimulating the same morphological and biochemical acclimation pathways usually activated by other mild environmental stresses, alongside the occurrence of eustress phenomena. Over extended periods, this stimulus could foster adaptive changes, enabling plants to thrive in space.

## 1. Introduction

Microgreens, small plants harvested prematurely at the stage of full cotyledon expansion or at the first emergence of true leaves, have seen a surge in popularity during the 2020s [1]. The advantages over mature plants include a shorter growth cycle, small dimensions, appealing colors, and an abundance of phytonutrients with profiles that vary significantly across species [2]. Harvested soon after sprouting, microgreens accumulate a higher concentration of phytonutrients compared to their adult counterparts, primarily to protect themselves from diseases and predators in a very delicate early stage of growth. For these reasons, microgreens have forcefully entered the list of candidates for space farming [3]. Their rich flavors and unique nutrient profiles may enhance appetite and support the physiological balance of crew members [4]. Furthermore, the ease of cultivation and vibrant colors make microgreens suitable for horticultural therapy applications [5].

Despite the numerous challenges posed by the microgravity environment for space farming, various technologies and growth systems are being proposed or developed to facilitate successful microgreens cultivation in space [6,7]. For instance, NASA scientists from the crop food production research team have performed several parabolic flights inducing Martian (3/8 g) and Lunar (1/6 g) gravity levels, during which they conducted different growing and harvesting tests on microgreens [8].

However, one of the most critical factors in space that could hinder plant growth and development, especially during the initial phases of seedling establishment, is ionizing radiation [9]. The space radiation environment consists of a diverse array of ion species spanning a wide range of energies. The primary components of galactic cosmic rays include high-energy protons, alpha particles, and heavy ions (like ^56^Fe and ^12^C) [10].

The impact of space radiation on plants has received considerably less attention in the scientific community compared to altered gravity, likely because most experiments conducted so far have typically taken place within low Earth orbit (LEO) facilities and involve brief exposure. As a result, the doses administered are often insufficient to cause significant changes in plants, which tend to exhibit greater resilience compared to mammals. To date, however, scientific research on the effects of radiation on plants serves a dual purpose. On the one hand, it aims to elucidate the impacts of space radiation and establish criteria for radiation shielding in future planet colonies; on the other hand, it has been observed that low doses of acute or chronic exposure to various types of ionizing radiation can induce eustress phenomena (also defined to as “radiation hormesis”) in plants [11,12]. Eustress means beneficial stress; when applied to humans, it encompasses psychological, physical, or biochemical benefits [13]. In plants, eustress is associated with positive phenomena (sometimes induced by ionizing radiation) that may stimulate growth and enhance the production of antioxidants and phytonutrients, serving as therapeutic countermeasures for human radiation protection.

In 2006, one of the first studies on this topic highlighted that antioxidant compounds obtained directly from consuming fresh vegetables, rather than through dietary supplements, have been effective in reducing several negative impacts of radiation-induced cellular oxidative stress [14]; moreover, growing plants on the International Space Station (ISS) has demonstrated benefits for alleviating psychological stress and depression linked to extended stays in space.

The responses of plants to ionizing radiation are often specific to the different species and cultivars [12] as well as to the particular phenological stage of exposure to the radiation source (e.g., with seeds, seedlings, or leaves). For instance, in previous experiments on eye bean irradiated at the dry seed stage with carbon and titanium ions, we observed a completely different response of the same species. While carbon ion exposure reduced germination, surviving seedlings did not show impairment in morpho-anatomical development, exhibiting only slowed growth compared to titanium-irradiated plants [9]. In another study, chard irradiated with the same heavy ions showed that control and carbon-ion-irradiated plants showed a physiological performance superior to that of titanium-ion-irradiated plants in terms of the content of pigments, PSII photochemical efficiency, and bioactive compounds production [10].

Therefore, investigating plant responses to specific ions at appropriate doses is essential for assessing radiosensitivity and identifying the optimal ion–dose combinations to induce eustress in target species. Indeed, plants could potentially acclimate to altered-radiation environments by adjusting stress-related signaling pathways, thereby enhancing resilience, similar to responses observed with other mild stresses (water and salt stress) on Earth [15]. Over extended periods, this approach could foster adaptive changes, enabling plants to thrive in space [16,17].

However, there is still a knowledge gap concerning the effects of radiation on plants related to radio-sensitivity/resistance mechanisms. Additionally, efficient and rapid methods for selecting new species subjected to different types of ionizing radiation for space farming are required.

Due to the limited opportunities to expose plants to chronic radiation and the poor space fidelity of available facilities, experiments using specific heavy ions at defined acute doses are considered an essential preliminary approach in space biology. These experiments aim to investigate the radio-resistance of various species and assess their potential for cultivation in space [9].

For this purpose, we analyzed the responses of four species grown as microgreens to iron (^56^Fe) and carbon (^12^C) heavy ions. Dry seeds of *Ocimum basilicum* L. (basil), *Eruca vesicaria* subsp. *sativa* (Mill.) Thell. (rocket), *Raphanus raphanistrum* subsp. *sativus* (radish), and *Lepidium sativum* (L.) Domin (cress) were irradiated with doses of 0.3, 1, 10, 20, and 25 Gy of the two ions. After irradiation, seeds were germinated and microgreens cultivated under controlled conditions to investigate the morphological responses to radiation along with the production of photosynthetic pigments (chlorophyll and carotenoids) to explore the following: (i) the pattern of radiosensitivity/resistance, if any; and (ii) whether the morphological parameters, in relation to pigment content, could serve as a feasible method for screening radiation responses across microgreens species. If eustress phenomena are confirmed, it may be possible to reconsider the need for complete radiation shielding in future Martian or lunar greenhouses.

## 2. Results

### 2.1. Germination

**Figure 1:**
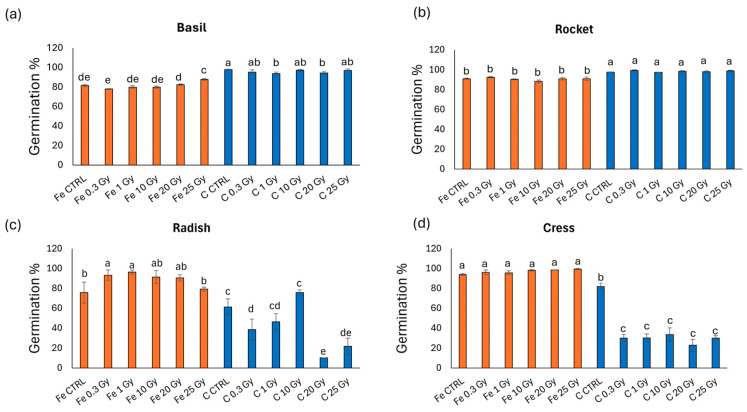
Germination % of (**a**) basil (*O. basilicum*), (**b**) rocket (*E. vesicaria*), (**c**) radish (*R. raphanistrum*), and (**d**) cress (*L. sativum*) seeds irradiated with Fe and C ions at the doses of 0.3, 1, 10, 20, 25 Gy, and CTRL. Mean values and corresponding standard errors are reported; different letters indicate significantly different values according to Duncan’s test (*p* < 0.05).

Figure 1 shows the germination percentage of the four different species grown as microgreens. Notably, the four species exhibited different trends: basil and rocket presented higher germination % in the C-exposed microgreens, while radish and cress had higher germination % in the Fe-exposed microgreens. Specifically, basil (Figure 1a) exhibited an enhancement in germination compared to the control (CTRL) in Fe-exposed microgreens only at 25 Gy, while C-exposed microgreens presented a reduction compared to the control at the doses of 1 and 20 Gy. Rocket (Figure 1b) did not show statistically significant differences among doses within each ion. Radish (Figure 1c) followed a more varied trend, with an increase in germination at Fe 0.3 and 1 Gy (compared to the control), and a decrease at C 0.3, 20, and 25 Gy, with a particularly marked reduction in germination at C 20 Gy (83% reduction). Cress (Figure 1d) showed no differences among doses in Fe-exposed microgreens and a severe reduction in all C-exposed microgreens compared to the control, with no statistically significant differences among the doses.

### 2.2. Biometric and Morphological Analyses

Table 1 summarizes the effects of ion (I), dose (D), and their interaction (I × D) on the biometric and morphological characteristics of basil. Iron ions significantly stimulated both root and leaf lengths (*p* < 0.05) as well as the microgreen area (*p* < 0.001), while C ions primarily promoted total microgreen elongation (*p* < 0.001). Different doses of irradiation significantly affected root length and microgreen area (*p* < 0.01), with distinct trends observed: root length increased from the control to 25 Gy, whereas microgreen area decreased at 0.3, 1, and 25 Gy and expanded at 10 Gy compared to the control. The interaction between the main factors (I × D) was significant only for the microgreen area (*p* < 0.001), revealing a divergence between Fe and C treatments. Specifically, Fe exhibited a reduction in area compared to the control at 0.3 and 1 Gy, while C showed a reduction compared to the control at all doses but with no statistically significant differences across all doses, consistently presenting the greatest reduction relative to all Fe doses.

Table 2 reports on the effects of ion (I), dose (D), and their interaction (I × D) on the biometric and morphological characteristics of rocket. Fe ions significantly stimulated both the root and microgreen area (*p* < 0.001), while C ions primarily promoted microgreen and leaf elongation (*p* < 0.01). Different doses of irradiation significantly affected only the leaf length (*p* < 0.05), showing a reduction compared to CTRL from 1 Gy to 25 Gy, with a more pronounced decrease at 1 and 20 Gy. The interaction between the main factors (I × D) was significant for all parameters with *p* < 0.01, except for the root length with *p* < 0.05. Regarding root length, differences were observed only between Fe and C ions, with Fe showing higher values. The elongation of microgreens and their area followed a similar trend, with higher values in Fe and lower in C across all doses. However, within the C treatments, increases in microgreen length were noted at 1 Gy and 20 Gy compared to the control. Leaf elongation was enhanced only in the C control and C 0.3 Gy treatments while no statistically significant differences were observed among the other treatments. Microgreen area, instead, was enhanced at 1 and 20 Gy in Fe-exposed microgreens, while C-exposed microgreens showed a reduction compared to the control at all doses but no statistically significant differences across all doses, consistently presenting the greatest reduction relative to all Fe doses.

Table 3 reports on the effects of ion (I), dose (D), and their interaction (I × D) on the biometric and morphological characteristics of radish. Fe ions significantly stimulated all parameters with *p* < 0.001 for the root and microgreen length, and *p* < 0.01 for the leaf length and microgreen area. Different doses of irradiation significantly affected all parameters with a different degree of significance (*p* < 0.05 for the root length; *p* < 0.001 for the microgreen length and area; and *p* < 0.01 for the leaf length), and with distinct trends observed: root length increased at 1 Gy and particularly at 10 Gy, while decreased at 25 Gy compared to CTRL. Major decreases at 25 Gy (along with 20 Gy) were also evident in the leaf length and microgreen area. Microgreen length showed a significant reduction only at 20 Gy.

The interaction between the main factors (I × D) was significant for all parameters except for the root length. Microgreen length (*p* < 0.01) reduced in the C-ion treatment at 1 Gy and particularly at 20 Gy (86% reduction compared to CTRL). Similarly, leaf length and microgreen area (*p* < 0.001) also showed the highest reduction at 20 Gy in the C-ion treatment, with only slightly significant differences observed among other treatments.

Table 4 reports on the effects of ion (I), dose (D), and their interaction (I × D) on the biometric and morphological characteristics of cress. In this species, Fe ions significantly stimulated all parameters (*p* < 0.001 for the root and microgreen length; *p* < 0.05 for the leaf length and microgreen area). Different doses of irradiation significantly affected only root length (*p* < 0.001), with a reduction observed at 20, 25, and particularly at 0.3 Gy compared to CTRL. The interaction between the main factors (I × D) was significant only for the root length (*p* < 0.001) and microgreen length (*p* < 0.05), revealing a divergence between Fe and C treatments. Notably, Fe exhibited a reduction in both root and microgreen length at 25 Gy. This reduction was not observed with C ions, where 0.3 Gy was the treatment to develop the shortest roots, and 1 Gy was the treatment to develop the shortest length.

**Figure 2:**
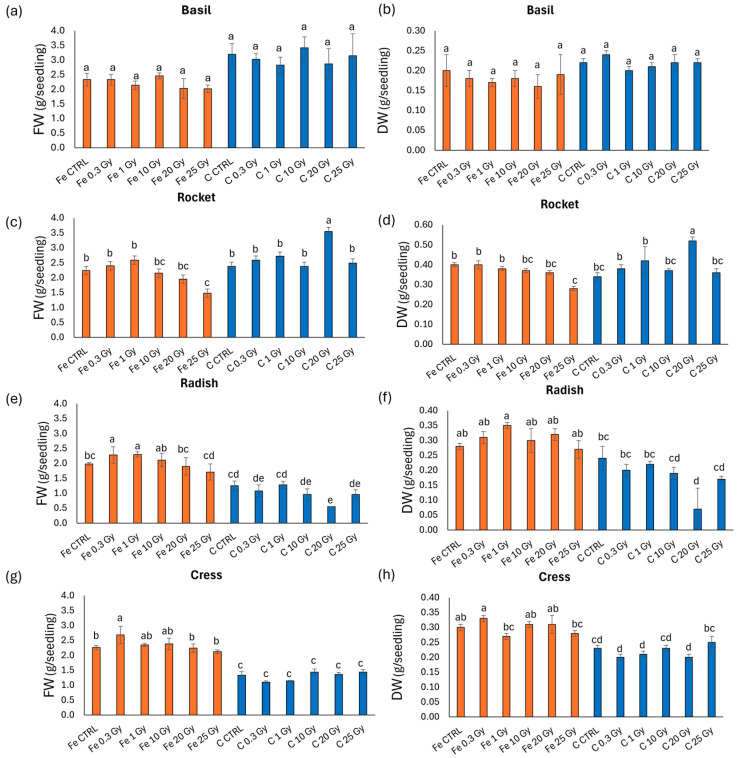
Fresh and dry biomass (g/seedling) of (**a**,**b**) basil (*O. basilicum*), (**c**,**d**) rocket (*E. vesicaria*), (**e**,**f**) radish (*R. raphanistrum*), and (**g**,**h**) cress (*L. sativum*) microgreens whose seeds were irradiated with Fe and C ions at the doses of 0.3, 1, 10, 20, 25 Gy, and CTRL. Mean values and corresponding standard errors are reported; different letters indicate significantly different values according to Duncan’s test (*p* < 0.05).

Figure 2 illustrates the biomass in terms of fresh weight (FW) and dry weight (DW) of the four microgreens species. Notably, the species did not follow the same trend of variation. Basil (Figure 2a,b) showed no statistically significant differences among treatments for either FW or DW. In rocket (Figure 2c,d), increases in both FW and DW were observed at C 20 Gy, while reductions occurred at Fe 25 Gy compared to the non-irradiated controls. Both radish (Figure 2e,f) and cress (Figure 2g,h) tended to produce heavier microgreens when irradiated with Fe compared to C ions. Specifically, radish FW and DW increased at 0.3 and 1 Gy in the Fe-ion treatment and decreased at 20 Gy in the C-ion treatment compared to the control. Cress exhibited similar weight increases at Fe 0.3 Gy compared to the control (though not significant in DW), with the lowest values observed in all C-ion-exposed microgreens.

### 2.3. Chlorophyll and Carotenoids’ Concentrations

**Figure 3:**
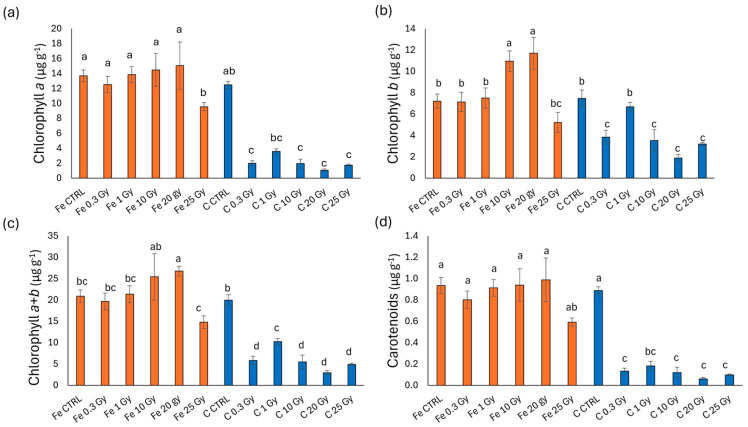
Chlorophyll *a* (**a**), chlorophyll *b* (**b**), chlorophyll *a* + *b* (**c**), and carotenoids (**d**) content of basil (*O. basilicum*) microgreens irradiated with Fe and C ions at the doses of 0.3, 1, 10, 20, 25 Gy, and CTRL. Mean values and corresponding standard errors are reported; different letters indicate significantly different values according to Duncan’s test (*p* < 0.05).

Figure 3 presents the photosynthetic pigment content in terms of chlorophyll *a*, *b*, *a* + *b*, and carotenoids in basil microgreens irradiated with Fe and C ions. As depicted in the figure, Fe-ion-irradiated microgreens display a higher pigment content compared to those irradiated with C ions. In detail, chlorophyll *a* (Figure 3a) reduced compared to CTRL only in Fe at 25 Gy (30% reduction), whereas reductions in C-irradiated microgreens were observed across all doses and were more pronounced (averaging between 71 and 91%). Chlorophyll *b* (Figure 3b), chlorophyll *a* + *b* (Figure 3c), and carotenoids (Figure 3d) displayed a similar trend with minor variations. More specifically, in contrast to chlorophyll *a*, chlorophyll *b* levels increased in Fe-irradiated treatments at 10 and 20 Gy compared to the controls and the other doses. For carbon-ion treatments, chlorophyll *b* values were generally higher compared to chlorophyll *a*. C 0.3, 10, 20, and 25 Gy microgreens presented significantly lower values than all Fe-ion treatments but Fe 25 Gy. This trend of variation persisted in total chlorophyll (*a* + *b*) and carotenoids contents (Figure 3c,d).

**Figure 4:**
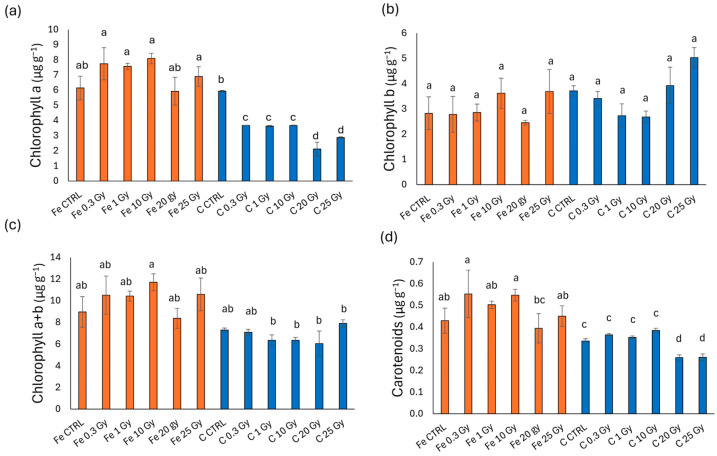
Chlorophyll *a* (**a**), chlorophyll *b* (**b**), chlorophyll *a* + *b* (**c**), and carotenoids (**d**) content of rocket (*E. vesicaria*) microgreens irradiated with Fe and C ions at the doses of 0.3, 1, 10, 20, 25 Gy, and CTRL. Mean values and corresponding standard errors are reported; different letters indicate significantly different values according to Duncan’s test (*p* < 0.05).

Figure 4 shows the photosynthetic pigment content in rocket microgreens irradiated with Fe and C ions. For chlorophyll *a* (Figure 4a), the Fe-treated samples showed values comparable to the control, while in the C-treated samples, there was a marked decrease for all doses compared to the control, with the lowest chlorophyll *a* content at doses of 20 and 25 Gy. Chlorophyll *b* (Figure 4b) did not show significant differences between ions or irradiation doses. Similarly, total chlorophyll (chlorophyll *a* + *b*, Figure 4c) showed no significant differences in Fe-irradiated microgreens, though a significant reduction was observed at doses from 1 to 25 Gy with C ions compared to the highest content recorded with Fe at 10 Gy, with all other values being intermediate. Carotenoids (Figure 4d) were stimulated in the Fe treatment at doses of 0.3 and 10 Gy, while a sharp reduction was observed with carbon at all doses, particularly at the highest doses of 20 and 25 Gy (reduction by 23% compared to the C control).

**Figure 5:**
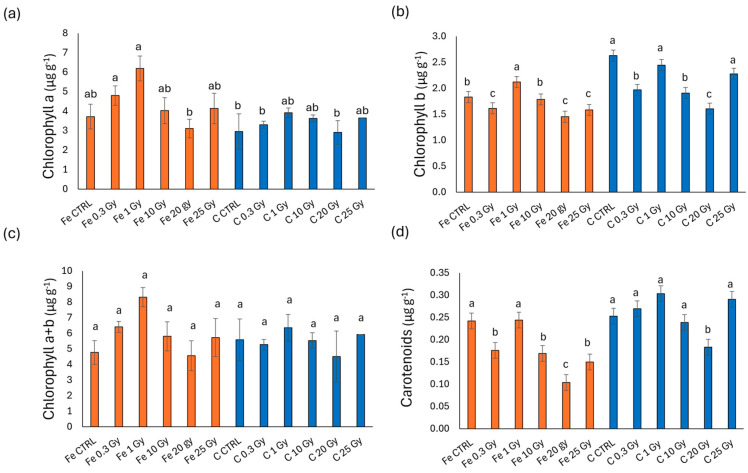
Chlorophyll *a* (**a**), chlorophyll *b* (**b**), chlorophyll *a* + *b* (**c**), and carotenoids (**d**) content of radish (*R. raphanistrum*) microgreens irradiated with Fe and C ions at the doses of 0.3, 1, 10, 20, 25 Gy, and CTRL. Mean values and corresponding standard errors are reported; different letters indicate significantly different values according to Duncan’s test (*p* < 0.05).

Figure 5 shows the photosynthetic pigment content in radish microgreens irradiated with Fe and C ions. As illustrated, only slight differences are observed between microgreens irradiated with Fe ions and those irradiated with C ions. Chlorophyll *b* (Figure 5b), on the other hand, showed an increase compared to the control at a dose of 1 Gy with iron ions but a decrease compared to the control at the highest doses (20 and 25 Gy); this trend is not observed with carbon ions, where at 1 Gy— as well as at 25 Gy—the values were comparable to the control. No significant differences were found for total chlorophyll (Figure 5c), while for carotenoids (Figure 5d) a decrease was observed compared to the control with Fe ions at 0.3, 10, 20, and 25 Gy, with the most marked reduction at 20 Gy; for C ions, this reduction was evident only at 20 Gy in C-irradiated samples, still significantly higher than Fe 20 Gy.

**Figure 6:**
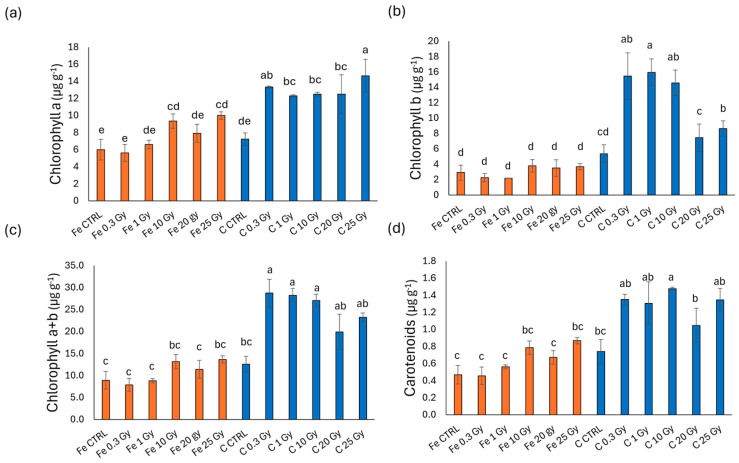
Chlorophyll *a* (**a**), chlorophyll *b* (**b**), chlorophyll *a* + *b* (**c**), and carotenoids (**d**) content of cress (*L. sativum*) microgreens irradiated with Fe and C ions at the doses of 0.3, 1, 10, 20, 25 Gy, and CTRL. Mean values and corresponding standard errors are reported; different letters indicate significantly different values according to Duncan’s test (*p* < 0.05).

Figure 6 shows the photosynthetic pigment content in cress microgreens irradiated with Fe and C ions. C-ion-irradiated microgreens of this species exhibited higher pigment levels than those irradiated with Fe ions. Interestingly, rather than decreasing with increased irradiation doses, in both ions, the pigment levels were raised in comparison to the control, which displayed the lowest values across all four parameters analyzed.

Chlorophyll *b* (Figure 6b) exhibited a slightly different trend. Fe-ion-treated microgreens showed the lowest levels overall, with no significant differences between the control and the irradiated doses. In C-treated samples, however, chlorophyll *b* increased significantly above the control level at lower doses (0.3, 1, and 10 Gy) and then decreased again at 20 and 25 Gy, even if remaining above the control at 25 Gy.

Total chlorophyll and carotenoids contents (Figure 6c,d) followed a trend more similar to that of chlorophyll *a*, with levels equal to or higher than the control across all irradiation doses for both Fe and C ions, with markedly higher levels observed in C treatments.

### 2.4. Hierarchical Clustering and Correlation Plot

**Figure 7:**
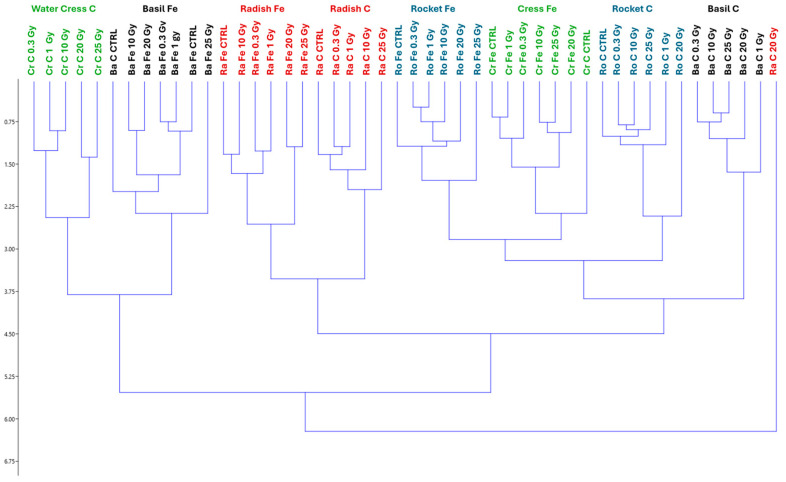
Hierarchical cluster analysis (HCA) of morphological traits and pigment content of basil (Ba), rocket (Ro), radish (Ra), and cress (Cr) microgreens whose seeds were irradiated with Fe and C ions at the doses of 0.3, 1, 10, 20, 25 Gy, and CTRL.

The hierarchical cluster analysis (Figure 7) illustrates the clustering of the four species irradiated with the two heavy ions. By cutting the dendrogram at the distance value of 4.5, according to the agglomeration schedule, three clusters appeared: the first on the right containing only radish irradiated with carbon ions at the dose of 20 Gy (Ra C 20 Gy); a second one on the left containing water cress (Cr) irradiated with carbon ions and basil irradiated with Fe ions; and a third, larger cluster encompassing all other treatments.

However, cutting the dendrogram at a distance of 3.5, eight clusters are identified, clearly separating each species and each type of heavy ion, with two exceptions: (i) Ra C 20 Gy remains isolated; and (ii) radish clusters together, without differentiating between Fe- and C-irradiated ions.

Many positive and negative correlations at different significant levels (*p* < 0.001 ***, *p* < 0.01 **, *p* < 0.05 *) were found between morphological biometric traits (germination %, G; fresh weight, FW; dry weight, DW; microgreen area, MA) and pigment content (chlorophyll *a*, Chla; chlorophyll *b*, Chlb; chlorophyll *a* + *b*, Chlab; carotenoids, Car).

**Figure 8:**
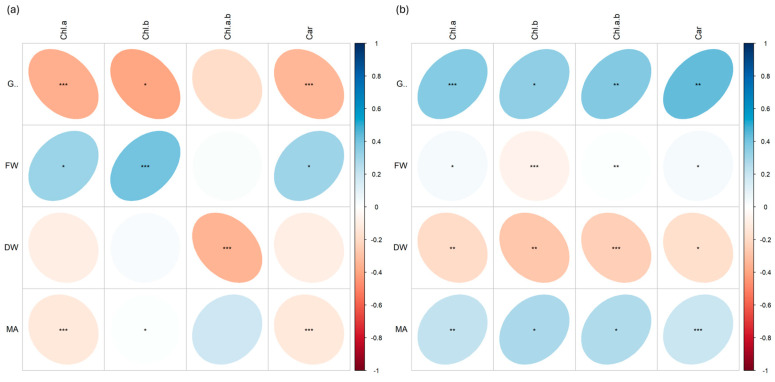
Spearman’s rank correlation coefficients between pairs of biometric traits (germination %, G; fresh weight, FW; dry weight, DW; microgreen area, MA) and pigment content (chlorophyll *a*, Chl.a; chlorophyll *b*, Chl.b; chlorophyll *a* + *b*, Chl.a.b.; carotenoids, Car.) in basil (*O. basilicum*) microgreens irradiated with Fe (**a**) and C (**b**) ions at the doses of 0.3, 1, 10, 20, 25 Gy, and CTRL. Positive and negative correlations are shown; *; **, and *** are significant at *p* < 0.05, 0.01, and 0.001, respectively.

In basil (Figure 8), strong negative correlations were found between germination and pigment content in plants irradiated with Fe ions (Figure 8a), while strong positive correlations were found between the same parameters in plants irradiated with C ions (Figure 8b). Similar differences also appear for FW, which was positively correlated with almost all pigments in the Fe treatment but not in the C treatment; in addition, MA was negatively correlated with Chla and Car and positively with Chlb and Chlab in plants irradiated with C ions, while it was always positively correlated in plants irradiated with Fe ions.

**Figure 9:**
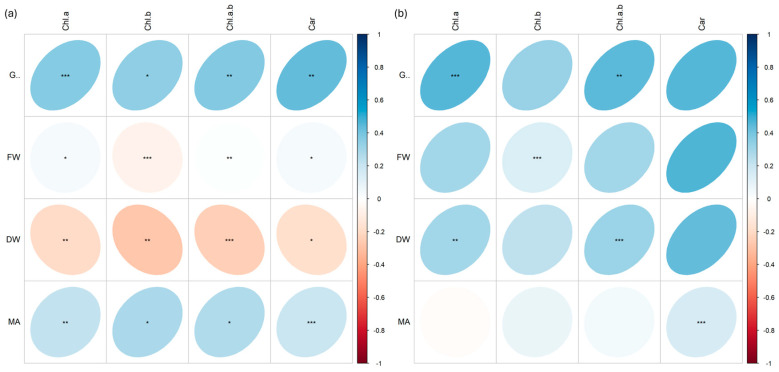
Spearman’s rank correlation coefficients between pairs of biometric traits (germination, G; fresh weight, FW; dry weight, DW; microgreen area, MA) and pigment content (chlorophyll *a*, Chl.a; chlorophyll *b*, Chl.b; chlorophyll *a* + *b*, Chl.a.b.; carotenoids, Car.) in rocket (*E. vesicaria*) microgreens irradiated with Fe (**a**) and C (**b**) ions at the doses of 0.3, 1, 10, 20, 25 Gy, and CTRL. Positive and negative correlations are shown; *; **, and *** are significant at *p* < 0.05, 0.01, and 0.001, respectively.

In rocket (Figure 9), while plants treated with carbon (Figure 9b) showed all positive correlations between the data, those with iron showed negative correlations (more or less strong) between FW and Chlb, and between DW and all pigments (Figure 9a).

**Figure 10:**
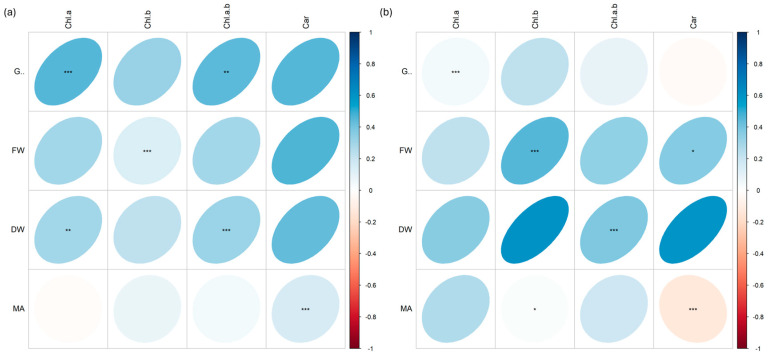
Spearman’s rank correlation coefficients between pairs of biometric traits (germination, G; fresh weight, FW; dry weight, DW; microgreen area, MA) and pigment content (chlorophyll *a*, Chl.a; chlorophyll *b*, Chl.b; chlorophyll *a* + *b*, Chl.a.b.; carotenoids, Car.) in radish (*R. raphanistrum*) microgreens irradiated with Fe (**a**) and C (**b**) ions at the doses of 0.3, 1, 10, 20, 25 Gy, and CTRL. Positive and negative correlations are shown; *; **, and *** are significant at *p* < 0.05, 0.01, and 0.001, respectively.

In radish (Figure 10), all parameters were positively correlated except for the correlation between MA and Car, which showed a strong negative correlation in plants treated with C ions (Figure 10b).

**Figure 11:**
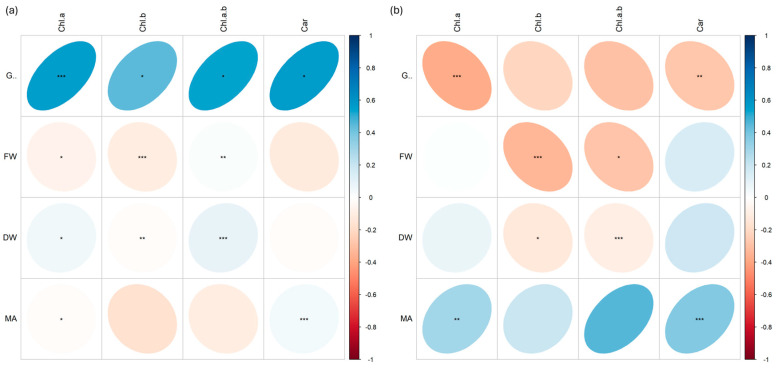
Spearman’s rank correlation coefficients between pairs of biometric traits (germination, G; fresh weight, FW; dry weight, DW; microgreen area, MA) and pigment content (chlorophyll *a*, Chl.a; chlorophyll *b*, Chl.b; chlorophyll *a* + *b*, Chl.a.b.; carotenoids, Car.) in cress (*L. sativum*) microgreens irradiated with Fe (**a**) and C (**b**) ions at the doses of 0.3, 1, 10, 20, 25 Gy, and CTRL. Positive and negative correlations are shown; *; **, and *** are significant at *p* < 0.05, 0.01, and 0.001, respectively.

In cress (Figure 11), more marked differences were observed among the data in both plants irradiated with Fe and C ions. Positive correlations were found between germination % and pigment content in plants irradiated with Fe ions (Figure 11a), while strong negative correlations were found between the same parameters in plants irradiated with C ions, especially between germination and chlorophyll *a* and between germination and carotenoids (Figure 11b). Similar differences also appeared for DW, which was positively correlated with chlorophyll *b* and *a* + *b* in Fe but not in C, where the same correlation was negative.

## 3. Discussion

Different heavy ion types, delivered at varying doses, differentially influenced germination percentage, growth, biomass accumulation, and pigment content in the four species analyzed: basil, rocket, radish, and cress. The response of the four species to ionizing radiation was found to be highly species-specific. Ionizing radiation was often perceived by the microgreens as a mild stress, triggering morphological and biochemical acclimation pathways similar to those activated by other mild environmental stresses.

Previous studies on the effects of ionizing radiation on plants [12] have highlighted that radiation response is often highly specific to species and even to cultivars. Moreover, it is now understood that the impact of space-related factors, including radiation, depends greatly on the phenological stage at the time of irradiation and also on the developmental stage at the time of the analysis.

In this study, all species were irradiated at the stage of dry seeds and subsequently grown to the microgreens stage, cultivated under the same environmental conditions. Notably, basil and rocket displayed a more similar response pattern compared to radish and cress. Specifically, in both basil and rocket, the germination percentage remained mostly unchanged after the exposure to both Fe and C ions compared to the controls. In radish, irradiation with Fe promoted germination at low doses (i.e., 0.3 and 1 Gy), while C irradiation reduced germination % at very low (0.3 Gy) and very high doses (20 and 25 Gy). In cress, Fe irradiation did not induce any changes in germination. In contrast, C ions reduced germination independently from the dose. There is not much literature available on the effects of heavy ions on plants; however, earlier research indicates that C ions had no significant effect on the germination of mung bean but, in accordance with our findings on radish and cress, C ions did reduce germination in tobacco, eye bean, and sorghum [9,18,19]. Other studies on X-ray ionizing radiation [20] have shown that exposing seeds of *Solanum lycopersicum* L. and *Vigna radiata* L. to increasing doses (up to 50 Gy) does not affect their germination percentage. In contrast, as confirmed by our study, the exposure to high-LET radiation (heavy ions) can lead to reductions in germination and survival at specific doses, as observed in *Nicotiana tabacum* L. seeds subjected to carbon-ion irradiation, which induced chromosomal aberrations [21]. Similarly, in tomatoes irradiated with Ca ions at 25 Gy, germination rates decreased following the irradiation.

Even though data on ionizing radiation effects on plants within the field of space biology remain limited, it is known that abiotic stresses generally reduce germination rates [22]. Stress factors such as radiation, salinity, extreme temperatures, or drought can disrupt physiological processes in seeds, leading to slower or inhibited germination. In general, abiotic stresses tend to hinder plant development; however, under certain conditions, mild stress can induce an adaptive response by activating regulatory pathways, enabling plants to respond and adapt within their environments [22]. In this study, C-ion irradiation was probably sensed by radish and cress plants as a mild stress since germination and growth were not prevented but C-ion-exposed plants tended to significantly decrease in the levels of most analyzed morphological parameters (Figure 2; Table 1, Table 2, Table 3 and Table 4). The contrasting effects of C-ion versus Fe-ion irradiation in basil and rocket were also evident in biomass measurements (fresh and dry weight) and morphological parameters, with Fe irradiation leaving most parameters unchanged and C ions reducing most of the parameters measured.

The increased biomass in C-irradiated rocket could be linked to the enhanced length of microgreens and of its leaves, relative to the overall plant area. This more extensive length, probably allowing for better light absorption, thereby contributes to overall plant vigor. Additionally, the greater leaf length may enhance the plant’s ability to access light resources, which can further support growth and lead to increased fresh and dry weights [23].

C-exposed rocket probably invested more energy into producing new biomass rather than merely sustaining existing structures. This strategy may be an acclimation response to improve survival and competitiveness in the growing environment, ultimately resulting in improved yields for the C-irradiated rocket.

The situation reverses concerning pigments, with again a clear distinction between the four species. This time, however, basil and rocket tended to have lower chlorophyll and carotenoids levels in plants exposed to C ions compared to Fe ions, whereas cress pigments remained unchanged between treatments or even increased under Fe-ion exposure (Figure 3, Figure 4, Figure 5 and Figure 6). In agreement with our results, De Micco et al. [9] found eye bean seedlings had a lower value of photosynthetic pigments (chlorophylls and carotenoids) in carbon-exposed seedlings. This lower value determined a simultaneous reduction in the light harvesting capacity, consequently limiting photosynthesis at later developmental stages (adult plants).

Based on the germination % and morphological analyses (especially leaf length), it might seem odd that rocket and basil developed fewer pigments. Different reasons could explain this phenomenon: (i) different resource allocation, meaning that these plants invest more resources in developing hypocotyls and may have less energy available for synthesizing chlorophylls and carotenoids [24]; (ii) environmental response to light intensity and quality or temperature—suboptimal parameters could limit pigment biosynthesis while not affecting growth metrics [25]; (iii) resource allocation—plants may allocate resources differently, prioritizing structural growth (e.g., leaf elongation) over secondary metabolite production like pigments [26].

These plants were all grown under the same conditions, subjected to the same environment; however, their requirements and resource use efficiency may not have been identical, as explained by Amitrano et al. [27] in their study on *Brassica oleracea* “Vertus” and *Raphanus raphanistrum* “saxa” microgreens for space farming.

In summary, the increased length of rocket under the C-ion treatment could have influenced the production of chlorophylls and carotenoids due to the need to balance resources and energy between different vital functions of the plant. Concerning the opposite response of radish and cress, these species seemed to show a better acclimation capacity than basil and rocket to carbon ions due to their higher photosynthetic pigment content.

A mild stress, such as exposure to certain ions or environmental changes, can trigger adaptive responses in plants that enhance photosynthetic pigment content [28]. This increment in pigments, particularly chlorophylls and carotenoids, likely serves as a protective mechanism. Chlorophylls are essential for capturing light energy, so improved chlorophyll production can help the plant maintain good photosynthetic rates under suboptimal conditions [29]. Carotenoids play a dual role: assisting in light absorption for photosynthesis and acting as antioxidants, protecting chlorophyll and other cellular components from oxidative damage caused by stress-induced reactive oxygen species (ROS).

Ionizing radiation is known to indirectly generate reactive oxygen species (ROS), which may damage cellular structures such as lipids, proteins, and DNA [28]. ROS can have mixed effects: at low levels, they act as signaling molecules, prompting cells to strengthen their antioxidant defenses; however, in high concentrations, they lead to oxidative stress, overwhelming the cell’s defenses and potentially causing cellular damage or death [30]. By promptly boosting their pigment profile, plants may improve their energy capture and buffering capacity against oxidative stress. This adaptive response not only aids in maintaining growth and metabolic function but also primes the plant to better tolerate further environmental stress, supporting resilience and survival [31].

Looking at correlations between morphological traits and pigment content (Figure 8, Figure 9, Figure 10 and Figure 11), it is possible to notice differences mostly related to the different species genotypes.

For instance, the negative correlation between dry weight and chlorophyll content in cress may indicate that, in these plants, higher chlorophyll production does not translate into a proportional increase in dry biomass. This is the case for *Glycine max* (soybean) under nitrogen deficiency [32] and *Arabidospis thaliana* under water stress [33], where an increased chlorophyll concentration has been observed as the plants maximize their photosynthetic capacity with limited resources. However, the biomass accumulation remains constrained as resources are diverted to stress response mechanisms rather than to growth, and this could be due to various factors, such as a greater allocation of resources toward pigment production rather than vegetative growth, or an adaptive response to stress that stimulates chlorophyll production to improve photosynthetic efficiency.

On the other hand, the positive correlation between dry weight and chlorophyll content in radish suggests that an increase in chlorophyll is associated with greater biomass. This may indicate that, in these plants, higher photosynthetic capacity leads to better growth and accumulation of biomass, potentially due to increased efficiency in light absorption and conversion of solar energy into organic matter. However other studies on different ionizing radiation types have found a conspicuous reduction in plant growth and biomass, inducing in irradiated plants a more compact plant architecture [34,35].

The differences in correlations may arise from genetic, physiological, or environmental factors specific to each species, influencing how each plant responds to abiotic factors.

In this study, major differences between species were due to the different genotypes and irradiation ions. In general, there was no common effect of irradiation doses across the different species nor a dose threshold for any parameter was found. This is confirmed by the multivariate analysis of all traits, which shows that the four species clustered according to the ion, regardless of dose, with the sole exception of radish irradiated with C ions at 20 Gy, which stands alone (Figure 7).

At 20 Gy, C-irradiated radish showed a decrease in morphological parameters and germination % without affecting pigment levels. This might be due to radiation-induced stress that interferes with cellular structures and growth-regulating pathways. This intermediate dose may disrupt cell division and elongation processes, impacting morphology and germination without severely altering pigment biosynthesis pathways, which can be more resilient. Similar results were found in *Phaseolus vulgaris* L. juvenile leaves and in *Brassica rapa* L. microgreens exposed to X-rays, where 10 Gy induced a decrease in leaf morphological parameters and was recognized as a threshold dose for these parameters [15].

However, in radish exposed to the dose of 25 Gy, “hormesis” probably occurred since this dose stimulated an adaptive response that promoted recovery or even enhanced certain processes. This could allow radish to activate repair mechanisms, restoring morphological parameters and germination rates while pigment production remains stable.

The different photosynthetic pigment patterns of species tested in our study indicate that each species may modulate the response to ionizing radiation, also regulating light interception for photosynthesis and plant growth.

The overall data confirm that the reaction of microgreens to ionizing radiation is highly species- and cultivar-specific and that exploring morphological parameters in relation with pigment content could be an accurate and quick way to perform a screening of radiation responses among microgreens species.

## 4. Conclusions

This study demonstrates that the effects of ionizing radiation on microgreens are highly species-specific, with significant variability observed in germination, growth, biomass accumulation, and pigment content among basil, rocket, radish, and cress. The findings indicate that the mild stress induced by ionizing radiation can trigger adaptive responses, including morphological and biochemical changes, akin to those activated by other abiotic stresses. Notably, the differential responses to carbon (C) and iron (Fe) ions suggest distinct mechanisms of stress perception and acclimation in the species analyzed.

Additionally, correlations between morphological traits and pigment content highlight the potential for using these parameters as quick and effective indicators of radiation-induced stress in plants.

Future research could build on these findings by investigating the underlying mechanisms of plant responses to ionizing radiation, for example going further into the investigation of photosynthetic pathways and secondary metabolic adaptations. Furthermore, exploring gene-level responses could uncover regulatory networks and genetic determinants of radiation tolerance, offering opportunities for breeding or engineering stress-resilient crops.

By combining physiological, biochemical, and genetic analyses, future studies can provide a more comprehensive understanding of plant resilience to ionizing radiation and inform strategies to optimize plant growth in challenging environments, such as space habitats or radiation-contaminated regions on Earth.

## 5. Materials and Methods

### 5.1. Plant Material and Experimental Design

The experiment involved the irradiation of dry seeds from four plant species with heavy ions, subsequently cultivated up to the microgreen stage. The species included the following:

1. *Ocimum basilicum* L. (basil);
2. *Eruca vesicaria* subsp. *sativa* (Mill.) Thell. (rocket);
3. *Raphanus raphanistrum* subsp. *sativus* (radish);
4. *Lepidium sativum* L. (L.) Domin (cress).

Five increasing doses of heavy ions (0.3 Gy; 1 Gy; 10 Gy; 20 Gy; 25 Gy) were applied using two types of ionizing radiation, Iron (^56^Fe) and Carbon (^12^C) ions. Non-irradiated seeds served as the control group to evaluate morphological and biochemical responses of the microgreens to different radiation types and doses.

### 5.2. Species Description

Basil (*O. basilicum*)

An annual herb of the Lamiaceae family, basil is characterized by large, green, aromatic leaves on square stems. Thriving in warm climates (20–30 °C), it produces essential oils rich in antioxidants and bioactive compounds with antibacterial, antifungal, and insecticidal properties. Traditionally used in medicine for respiratory and digestive ailments, it also shows promise as an anti-stress and anti-leukemia agent [36].

Rocket (*E. vesicaria*)

A Brassicaceae family member, rocket is an herbaceous plant with lobed leaves, which grows well in moderate temperatures (15–25 °C) and low-fertility soils. Rocket leaves are nutrient-rich, containing proteins, vitamin C, and minerals, making them valuable in dietary applications for fresh and cooked foods [37].

Radish (*R. raphanistrum*)

Radish is a biennial Brassicaceae plant known for its fleshy, peppery root, which is cultivated in temperate climates with an optimal temperature range of 15−25 °C. Rich in bioactive compounds like glucosinolates, flavonoids, and β-carotene, radish is studied for its potential in nutraceuticals due to its health-promoting phytochemicals [38].

Cress (*L. sativum*)

A fast-growing herb from the Brassicaceae family, cress is cultivated widely in temperate regions (10–25 °C). Its seeds have notable medicinal applications as a diuretic, tonic, and galactagogue. Approximately 24% of the seeds’ oil content is composed of essential fatty acids, primarily α-linolenic acid (32%) and linoleic acid (12%), contributing to its nutritional value [39].

### 5.3. Irradiation, Sowing, and Cultivation Procedures

Irradiation was performed at the GSI Helmholtzzentrum für Schwerionenforschung (Darmstadt, Germany) using heavy ion beams of Carbon (^12^C; 290 MeV/u, 13 keV/µm) and Iron (^56^Fe; 1550 MeV/(g/cm²)) at the doses of 0.3 Gy, 1 Gy, 10 Gy, 20 Gy, and 25 Gy. A non-irradiated control (CTRL) group was included. Seeds were then transferred to the Plant and Wood Traits Laboratory at the Department of Agricultural Sciences, University of Naples Federico II, for cultivation under controlled conditions. Due to the availability of carbon and iron beams at different times of the year at the GSI Institute, simultaneous irradiation with ^12^C and ^56^Fe could not be performed. Consequently, we introduced in this paper two different controls (one for ^12^C and one for ^56^Fe). Thus, any observed differences between the Fe and C controls may be attributed to variations in cultivation timing.

For each species, irradiated seeds were divided into eighteen pots (three replicates per dose for both ^12^C and ^56^Fe treatments) on an inert sponge substrate. The sponge was soaked in distilled water, and the pots were incubated in the dark at 24 °C in a climate-controlled chamber with a 12/12 h photoperiod. Day/night temperatures and relative humidity were set at 24/18 ± 1 °C and 60%/70%, respectively. After germination, seedlings were cultivated until day 14 (harvest at microgreens stage) under white LED light at 200 ± 20 µmol·m^2^·s^−1^ at 24 °C, and fertigated daily with a quarter-strength modified Hoagland solution described in detail in Amitrano et al. [32].

Microgreens were harvested by cutting the seedlings above the substrate level with scissors. For each container, 10 microgreens were selected for biometric analysis, while the remaining biomass was used for fresh and dry weight measurements and biochemical analysis.

### 5.4. Germination % and Morphological Analysis

Germination percentage was calculated by counting the formed microgreens and dividing them by the number of seeds sown, following the formula:

Germination % = (Number of seeds germinated/Number of seeds sown) × 100(1)

The following morphological parameters were evaluated at harvest:

1. Canopy height (cm);
2. Fresh and dry biomass of seedlings (g): fresh weight was measured immediately after harvest, and dry weight was recorded after drying samples at 60 °C until constant weight;
3. Root, hypocotyl, leaf length (cm), and area (cm^2^): ten microgreens were placed on a black sheet, photographed, and analyzed for length and area using ImageJ software version 1.42 (Rasband, W.S., U.S. NIH, Bethesda, MD, USA).

### 5.5. Biochemical Analysis of Chlorophyll and Carotenoids

At harvest, chlorophyll and carotenoids contents were quantified using a spectrophotometric method following the protocol by Lichtenthaler (1987) [40]. Approximately 0.1 g of each sample was ground in a mortar with pure acetone (100%) as the solvent, and the extracts were centrifuged at 5000 rpm for 5 min. The supernatant was transferred to glass cuvettes, and absorbance was measured using a Cary 100 UV–visible spectrophotometer (Agilent Technologies, Santa Clara, CA, USA) at 470 nm, 645 nm, and 662 nm to determine the concentrations of chlorophyll *a*, chlorophyll *b*, and carotenoids, respectively. The concentrations were expressed in µg g^−1^. The quantification of photosynthetic pigments was performed with spectrophotometric analysis, a widely adopted method that, while providing approximate values (if compared to HPLC), is favored for its simplicity, accessibility, and ability to ensure comparability with similar studies.

### 5.6. Statistical Analysis

The biometric and biochemical data were analyzed using SPSS^®^ 13 software (SPSS Inc., Chicago, IL, USA). Shapiro–Wilk and Kolmogorov–Smirnov tests were performed to verify the normality of the data, and an arcsine transformation was applied to percentage data. A two-way ANOVA was conducted to analyze the main effects of ion type and dose, as well as their interaction. Post hoc tests (Duncan and SNK) were performed with a significance level set at *p* < 0.05. For multivariate analyses, hierarchical cluster analysis (HCA) was performed using Past 3 statistical software. The paired group (UPGMA) and Euclidean distances were used for clustering. Results of the HCA are displayed as a tree-shaped dendrogram (Figure 7), where the horizontal distance between clusters represents data dissimilarities. Variables used as input for both types of multivariate analyses were standardized to zero mean and unit variance. Correlation plots (*corrplot* package with Spearman’s method) were performed using the R software environment for statistical computing and graphics (version 4.4.1), as reported in detail in Amitrano et al. [41], to correlate biochemicals (pigment content) with morphological (germination %, fresh weight, dry weight, and microgreen area) analyses.

## Figures and Tables

**Table 1:** Effect of ion type (I), dose (D), and their interaction (I × D) on the morphological traits of basil (*O. basilicum*) microgreens irradiated with Fe and C ions at the doses of 0.3, 1, 10, 20, 25 Gy, and CTRL. Mean values and corresponding standard errors are reported; different letters indicate significantly different values according to Duncan’s test (*p* < 0.05). NS: not significant; *, **, ***: significant at *p* < 0.05, 0.01, and 0.001, respectively.

Basil	Root Length (cm)	Microgreen Length (cm)	Leaf Length (cm)	Microgreen Area (cm^2^)
Ion (I)				
Fe	0.39 ± 0.16 a	1.05 ± 0.29 b	0.38 ± 0.19 a	0.77 ± 0.25 a
C	0.34 ± 0.18 b	1.18 ± 0.21 a	0.34 ± 0.22 b	0.34 ± 0.10 b
Dose (D)				
CTRL	0.32 ± 0.02 c	1.23 ± 0.23 a	0.36 ± 0.03 a	0.57 ± 0.03 b
0.3 Gy	0.33 ± 0.04 bc	1.13 ± 0.24 a	0.41 ± 0.01 a	0.50 ± 0.03 d
1 Gy	0.35 ± 0.02 bc	0.98 ± 0.22 a	0.32 ± 0.03 a	0.50 ± 0.02 d
10 Gy	0.39 ± 0.02 b	1.15 ± 0.03 a	0.37 ± 0.02 a	0.62 ± 0.03 a
20 Gy	0.37 ± 0.01 b	1.31 ± 0.09 a	0.31 ± 0.04 a	0.58 ± 0.03 b
25 Gy	0.44 ± 0.03 a	0.91± 0.08 a	0.39 ± 0.04 a	0.55 ± 0.01 c
I × D				
Fe CTRL	0.32 ± 0.01 a	1.33 ± 0.16 a	0.39 ± 0.03 a	0.82 ± 0.04 a
Fe 0.3 Gy	0.35 ± 0.02 a	1.16 ± 0.16 a	0.40 ± 0.02 a	0.65 ± 0.03 b
Fe 1 Gy	0.37 ± 0.03 a	0.83 ± 0.16 a	0.38 ± 0.03 a	0.64 ± 0.03 b
Fe 10 Gy	0.41 ± 0.02 a	1.00 ± 0.01 a	0.38 ± 0.03 a	0.90 ± 0.04 a
Fe 20 Gy	0.43 ± 0.03 a	1.16 ± 0.16 a	0.31 ± 0.04 a	0.87 ± 0.04 a
Fe 25 Gy	0.45 ± 0.03 a	0.83 ± 0.16 a	0.45 ± 0.02 a	0.75 ± 0.03 ab
C CTRL	0.31 ± 0.01 a	1.13 ± 0.13 a	0.32 ± 0.04 a	0.73 ± 0.01 b
C 0.3 Gy	0.31 ± 0.03 a	1.10 ± 0.15 a	0.41 ± 0.03 a	0.36 ± 0.02 c
C 1 Gy	0.33 ± 0.02 a	1.13 ± 0.12 a	0.26 ± 0.04 a	0.36 ± 0.01 c
C 10 Gy	0.38 ± 0.02 a	1.30 ± 0.05 a	0.36 ± 0.03 a	0.34 ± 0.01 c
C 20 Gy	0.31 ± 0.02 a	1.46 ± 0.03 a	0.32 ± 0.03 a	0.29 ± 0.01 c
C 25 Gy	0.43 ± 0.04 a	1.00 ± 0.01 a	0.34 ± 0.04 a	0.34 ± 0.01 c
Significance				
I	*	***	*	***
D	**	NS	NS	**
I × D	NS	NS	NS	***

**Table 2:** Effect of ion type (I), dose (D), and their interaction (I × D) on the morphological traits of rocket (*E. vesicaria*) microgreens irradiated with Fe and C ions at the doses of 0.3, 1, 10, 20, 25 Gy, and CTRL. Mean values and corresponding standard errors are reported; different letters indicate significantly different values according to Duncan’s test (*p* < 0.05). NS: not significant; *, **, ***: significant at *p* < 0.05, 0.01, and 0.001, respectively.

Rocket	Root Length (cm)	Microgreen Length (cm)	Leaf Length (cm)	Microgreen Area (cm^2^)
Ion (I)				
Fe	1.01 ± 0.45 a	0.46 ± 0.11 b	0.62 ± 0.14 b	0.92 ± 0.30 a
C	0.52 ± 0.20 b	0.92 ± 0.30 a	0.68 ± 0.15 a	0.46 ± 0.11 b
Dose (D)				
CTRL	0.76 ± 0.06 a	0.64 ± 0.03 a	0.69 ± 0.02 a	0.64 ± 0.03 a
0.3 Gy	0.71 ± 0.04 a	0.70 ± 0.03 a	0.69 ± 0.03 a	0.70 ± 0.03 a
1 Gy	0.75 ± 0.06 a	0.72 ± 0.04 a	0.62 ± 0.02 c	0.72 ± 0.04 a
10 Gy	0.79 ± 0.04 a	0.68 ± 0.03 a	0.64 ± 0.02 b	0.68 ± 0.03 a
20 Gy	0.78 ± 0.05 a	0.70 ± 0.04 a	0.62 ± 0.02 c	0.70 ± 0.04 a
25 Gy	0.82 ± 0.06 a	0.70 ± 0.03 a	0.63 ± 0.01 bc	0.70 ± 0.02 a
I × D				
Fe CTRL	0.97 ± 0.08 a	0.50 ± 0.02 c	0.72 ± 0.02 ab	0.77 ± 0.03 b
Fe 0.3 Gy	0.97 ± 0.05 a	0.45 ± 0.01 c	0.62 ± 0.03 c	0.95 ± 0.05 ab
Fe 1 Gy	1.03 ± 0.09 a	0.43 ± 0.02 c	0.63 ± 0.02 bc	1.01 ± 0.06 a
Fe 10 Gy	1.01 ± 0.07 a	0.46 ± 0.02 c	0.62 ± 0.03 c	0.89 ± 0.04 ab
Fe 20 Gy	1.00 ± 0.08 a	0.43 ± 0.01 c	0.60 ± 0.02 c	0.97 ± 0.06 a
Fe 25 Gy	1.09 ± 0.09 a	0.47 ± 0.02 c	0.61 ± 0.03 c	0.93 ± 0.04 ab
C CTRL	0.55 ± 0.05 b	0.77 ± 0.03 b	0.77 ± 0.03 a	0.50 ± 0.02 bc
C 0.3 Gy	0.46 ± 0.03 b	0.95 ± 0.05 ab	0.76 ± 0.03 ab	0.45 ± 0.01 c
C 1 Gy	0.47 ± 0.03 b	1.01 ± 0.06 a	0.62 ± 0.03 c	0.43 ± 0.02 c
C 10 Gy	0.56 ± 0.02 b	0.89 ± 0.04 ab	0.66 ± 0.02 bc	0.46 ± 0.02 c
C 20 Gy	0.55 ± 0.03 b	0.97 ± 0.06 a	0.64 ± 0.02 bc	0.43 ± 0.01 c
C 25 Gy	0.55 ± 0.03 b	0.93 ± 0.04 ab	0.66 ± 0.02 bc	0.47 ± 0.02 c
Significance				
I	***	***	***	***
D	NS	NS	*	NS
I × D	*	**	**	**

**Table 3:** Effect of ion type (I), dose (D), and their interaction (I × D) on the morphological traits of radish (*R. raphanistrum*) microgreens irradiated with Fe and C ions at the doses of 0.3, 1, 10, 20, 25 Gy, and CTRL. Mean values and corresponding standard errors are reported; different letters indicate significantly different values according to Duncan’s test (*p* < 0.05). NS: not significant; *, **, ***: significant at *p* < 0.05, 0.01, and 0.001, respectively.

Radish	Root Length (cm)	Microgreen Length (cm)	Leaf Length (cm)	Microgreen Area (cm^2^)
Ion (I)				
Fe	1.42 ± 0.87 a	2.90 ± 0.35 a	0.68 ± 0.63 a	1.20 ± 0.57 a
C	0.78 ± 0.42 b	2.05 ± 0.88 b	0.51 ± 0.57 b	1.07 ± 0.55 b
Dose (D)				
CTRL	1.02 ± 0.09 c	2.73 ± 0.15 ab	0.54 ± 0.10 c	1.23 ± 0.06 a
0.3 Gy	1.15 ± 0.09 c	2.68 ± 0.04 ab	0.79 ± 0.06 a	1.23 ± 0.06 a
1 Gy	1.19 ± 0.11 b	2.55 ± 0.16 b	0.64 ± 0.04 b	1.28 ± 0.07 a
10 Gy	1.35 ± 0.11 a	2.60 ± 0.24 ab	0.65 ± 0.12 b	1.20 ± 0.06 ab
20 Gy	1.16 ± 0.16 c	1.48 ± 0.26 c	0.38 ± 0.04 d	0.88 ± 0.07 b
25 Gy	0.91 ± 0.09 d	2.80 ± 0.16 a	0.34 ± 0.12 d	0.92 ± 0.05 b
I × D				
Fe CTRL	1.39 ± 0.15 a	2.96 ± 0.03 a	0.71 ± 0.11 a	1.29 ± 0.10 a
Fe 0.3 Gy	1.51 ± 0.13 a	2.96 ± 0.03 a	0.91 ± 0.11 a	1.38 ± 0.11 a
Fe 1 Gy	1.44 ± 0.13 a	2.96 ± 0.03 a	0.75 ± 0.10 a	1.45 ± 0.09 a
Fe 10 Gy	1.71 ± 0.19 a	2.73 ± 0.27 ab	0.70 ± 0.11 a	1.14 ± 0.10 a
Fe 20 Gy	1.31 ± 0.17 a	2.63 ± 0.18 ab	0.47 ± 0.10 ab	0.98 ± 0.08 b
Fe 25 Gy	1.15 ± 0.13 a	3.13 ± 0.40 a	0.52 ± 0.12 ab	0.94 ± 0.09 b
C CTRL	0.63 ± 0.04 a	2.50 ± 0.28 ab	0.57 ± 0.09 ab	1.17 ± 0.12 a
C 0.3 Gy	0.77 ± 0.05 a	2.40 ± 0.05 ab	0.66 ± 0.10 a	1.07 ± 0.10 a
C 1 Gy	0.96 ± 0.09 a	2.13 ± 0.31 b	0.53 ± 0.08 ab	1.11 ± 0.50 a
C 10 Gy	0.94 ± 0.09 a	2.46 ± 0.21 ab	0.60 ± 0.12 a	1.27 ± 0.11 a
C 20 Gy	0.63 ± 0.16 a	0.33 ± 0.33 c	0.07 ± 0.07 b	0.48 ± 0.06 c
C 25 Gy	0.66 ± 0.06 a	2.46 ± 0.29 ab	0.56 ± 0.11 ab	0.90 ± 0.10 b
Significance				
I	***	***	**	**
D	*	***	**	***
I × D	NS	**	*	*

**Table 4:** Effect of ion type (I), dose (D), and their interaction (I × D) on the morphological traits of cress (*L. sativum*) microgreens irradiated with Fe and C ions at the doses of 0.3, 1, 10, 20, 25 Gy, and CTRL. Mean values and corresponding standard errors are reported; different letters indicate significantly different values according to Duncan’s test (*p* < 0.05). NS: not significant; *, ***: significant at *p* < 0.05, and 0.001, respectively.

Cress	Root Length (cm)	Microgreen Length (cm)	Leaf Length (cm)	Microgreen Area (cm^2^)
Ion (I)				
Fe	0.75 ± 0.29 a	2.33 ± 0.20 a	0.55 ± 0.33 a	0.58 ± 0.16 a
C	0.40 ± 0.30 b	1.61 ± 0.24 b	0.48 ± 0.44 b	0.56 ± 0.21 b
Dose (D)				
CTRL	0.71 ± 0.04 ab	1.98 ± 0.08 a	0.51 ± 0.07 a	0.54 ± 0.04 a
0.3 Gy	0.51 ± 0.03 d	2.01 ± 0.02 a	0.51 ± 0.06 a	0.55 ± 0.03 a
1 Gy	0.72 ± 0.05 a	1.98 ±0.02 a	0.54 ± 0.06 a	0.57 ± 0.03 a
10 Gy	0.74 ± 0.04 a	2.05 ± 0.15 a	0.54 ± 0.06 a	0.62 ± 0.03 a
20 Gy	0.65 ± 0.04 b	1.81 ± 0.11 a	0.48 ± 0.07 a	0.53 ± 0.02 a
25 Gy	0.69 ± 0.03 b	2.00 ± 0.10 a	0.51 ± 0.08 a	0.58 ± 0.02 a
I × D				
Fe CTRL	0.85 ± 0.06 a	2.50 ± 0.01 a	0.64 ± 0.07 a	0.57 ± 0.03 a
Fe 0.3 Gy	0.69 ± 0.04 abc	2.43 ± 0.03 a	0.54 ± 0.05 a	0.57 ± 0.02 a
Fe 1 Gy	0.78 ± 0.05 ab	2.50 ± 0.01 a	0.58 ± 0.05 a	0.55 ± 0.02 a
Fe 10 Gy	0.77 ± 0.04 ab	2.30 ± 0.16 ab	0.57 ± 0.06 a	0.58 ± 0.02 a
Fe 20 Gy	0.72 ± 0.04 abc	2.06 ± 0.06 bcd	0.46 ± 0.05 a	0.56 ± 0.02 a
Fe 25 Gy	0.68 ± 0.06 bc	2.16 ± 0.08 bc	0.51 ± 0.06 a	0.57 ± 0.04 a
C CTRL	0.78 ± 0.03 ab	2.46 ± 0.17 a	0.38 ± 0.07 a	0.52 ± 0.04 a
C 0.3 Gy	0.33 ± 0.03 d	1.60 ± 0.10 def	0.47 ± 0.08 a	0.52 ± 0.03 a
C 1 Gy	0.67 ± 0.06 bc	1.46 ± 0.08 f	0.49 ± 0.07 a	0.58 ± 0.04 a
C 10 Gy	0.71 ± 0.04 abc	1.76 ± 0.14 cde	0.51 ± 0.07 a	0.65 ± 0.03 a
C 20 Gy	0.58 ± 0.04 c	1.56 ± 0.17 ef	0.51 ± 0.09 a	0.50 ± 0.03 a
C 25 Gy	0.70 ± 0.03 abc	1.83 ± 0.12 cde	0.52 ± 0.09 a	0.59 ± 0.03 a
Significance				
I	***	***	*	*
D	***	NS	NS	NS
I × D	***	*	NS	NS

## Data Availability

The raw data supporting the conclusions of this article will be made available by the authors on request.
